# Mitochondrial analysis of oribatid mites provides insights into their atypical tRNA annotation, genome rearrangement and evolution

**DOI:** 10.1186/s13071-021-04719-0

**Published:** 2021-04-23

**Authors:** Xue-Bing Zhan, Bing Chen, Yu Fang, Fang-Yuan Dong, Wei-Xi Fang, Qian Luo, Ling-Miao Chu, Rui Feng, Yan Wang, Xuan Su, Ying Fang, Jiao-Yang Xu, Ze-Tao Zuo, Xing-Quan Xia, Jie-Gen Yu, En-Tao Sun

**Affiliations:** 1 Department of Health Inspection and Quarantine, Wannan Medical College, Wuhu, Anhui Province 241002 People’s Republic of China; 2 Department of Pathology, Wannan Medical College, Wuhu, Anhui Province 241002 People’s Republic of China; 3 Department of Management Science, Wannan Medical College, Wuhu, Anhui Province 241002 People’s Republic of China; 4 College of Life Science, the Provincial Key Lab of the Conservation and Exploitation Research of Biological Resources in Anhui, Anhui Normal University, Wuhu, Anhui Province 241000 People’s Republic of China

**Keywords:** Oribatid mites, Mitochondrial genome, TRNA re-annotation, Phylogeny

## Abstract

**Background:**

The mitochondrial (mt) genomes of Sarcoptiformes mites typically contain 37 genes. Although the loss of genes is rare in Sarcoptiformes mite mitogenomes, two of the six previously reported oribatid mites (Acariforms: Sarcoptiformes) are reported to have lost parts of their tRNA genes. To confirm whether the tRNA genes were indeed lost and whether the loss is universal, we re-annotated the available oribatid mite sequences and sequenced the mitogenome of *Oribatula sakamorii*.

**Methods:**

The mitogenome of *O*. *sakamorii* was sequenced using an Illumina HiSeq sequencer. The mt tRNA gene was annotated using multi-software combined with a manual annotation approach. Phylogenetic analyses were performed using the maximum likelihood and Bayesian inference methods with concatenated nucleotide and amino acid sequences.

**Results:**

The mitogenomes of *O*. *sakamorii* contained 37 genes, including 22 tRNA genes. We identified all mt tRNA genes that were reported as “lost” in *Steganacarus magnus* and *Paraleius leontonychus* and revealed certain atypical tRNA annotation errors in oribatid mite sequences. Oribatid mite mitogenomes are characterized by low rates of genetic rearrangement, with six or seven gene blocks conserved between the mitogenome of all species and that of ancestral arthropods. Considering the relative order of the major genes (protein-coding genes and rRNAs), only one or two genes were rearranged with respect to their positions in the ancestral genome. We explored the phylogenetic relationships among the available oribatid mites, and the results confirmed the systematic position of *Hermannia* in the Crotonioidea superfamily. This was also supported by the synapomorphic gene-derived boundaries.

**Conclusions:**

The tRNA “lost” phenomenon is not universal in oribatid mites. Rather, highly atypical secondary structure of the inferred mt tRNA genes made them unidentifiable using a single type of tRNA search program. The use of multi-software combined with a manual annotation approach can improve the accuracy of tRNA gene annotation. In addition, we identified the precise systematic position of *Hermannia* and validated that Astigmata is nested in Oribatida.

**Graphic Abstract:**

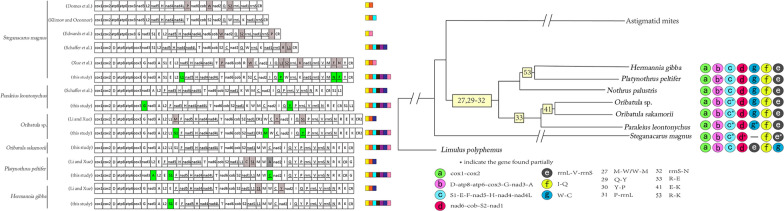

**Supplementary Information:**

The online version contains supplementary material available at 10.1186/s13071-021-04719-0.

## Background

Oribatida constitutes one of the two suborders of Sarcoptiformes. It comprises 56 superfamilies and more than 16,000 species. More than half (62%) of the Oribatida mite species are oribatid mites, whereas the rest (38%) are astigmatid mites [1]. Oribatid mites form an extremely diverse group of mites comprising five supercohorts: the most primitive Palaeosomatides, the early-derived Enarthronotides and Parhyposomatides, and the mid-to-highly derived Mixonomatides and Desmonomatides [2]. The supercohort Desmonomatides comprises three cohorts: Nothrina, Brachypylina and Astigmata. The precise relationship between oribatid and astigmatid mites is controversial. Based on morphological characteristics, there are three widely established hypotheses: (1) oribatid mites originated from astigmatid mites [3], (2) both Oribatida and Astigmata are monophyletic sister groups [4–6], and (3) astigmatid mites originated from oribatid mites [7–10]. Two different classification hypotheses have been proposed for oribatid mites (family Hermaniidae). Subías et al. considered Hermaniidae to be members of the superfamily Nanhermmanioidea as part of the Cohort Brachypylina based on their morphological characteristics [11]. Norton and Behan-Pelletier suggested that Hermaniidae should be included in the Cohort Nothrina superfamily Crotonioidea [12]. When species classification is not accurate or confirmed and is only based on morphological observation, molecular data are often used to clarify evolutionary and taxonomic issues [13–16]. It is necessary to study complete mitogenomes to assess the phylogenetic relationships among oribatid mites.

The mitogenome of Sarcoptiformes typically contains a conserved set of 37 genes. Of these, 13 are protein-coding genes (PCGs), 2 rRNAs genes (*rrnL* and *rrnS*) and 22 transfer RNA (tRNA) genes. Accurate gene annotation is important because mitogenomes may contain valuable phylogenetic information based on their gene orders. However, the identification of tRNAs lacking one or both arms or containing base mismatches in the stems is difficult. These tRNAs may be missed during the prediction process [17]. The mitogenome sequences of six oribatid mite species (order Sarcoptiformes) from two supercohorts (Mixonomatides and Desmonomatides) and six families (Phthiracaridae, Crotoniidae, Hermaniidae, Nothridae, Oribatulidae and Scheloribatidae) have been reported previously. In 2008, the first complete mitogenome sequence of an oribatid mite, *Steganacarus magnus*, was originally reported by Domes et al. to demonstrate a substantial loss of tRNAs (only 6 of 22 present) [18]. In 2009, Klimov and Oconnor retrieved *trnK* and re-annotated two tRNAs [(−)-*trnW*—> (+)-*trnS2*, (−)-*trnS2*—> (+)-*trnW*] based on minimum free energy (MFE) calculations. They refrained from considering the position of *trnP*, as followed by Domes et al. as well in 2008 [19]. In 2011, Edwards et al. used tRNAscan-SE [20] sequence alignments and manually inspected three of the missing tRNA genes (*trnG*, *trnS1* and *trnE*), based on which they altered the position of *trnP* [21]. In 2018, Schäffer et al. re-annotated the tRNA genes in *S. magnus* and identified 12 tRNA genes. They also sequenced the mitogenome of a new oribatid mite, *Paraleius leontonychus*, and observed that it lacked two tRNA genes (*trnG* and *trnY*). They also considered that tRNA loss occured in oribatid mites (order Sarcoptiformes) [17]. Xue et al. did not support the loss of mt tRNA genes in Sarcoptiformes mites, including oribatid mites. They retrieved the sequences of all 16 tRNA genes that were initially reported to be “lost” in *S*. *magnus* [22]. Using tRNAscan-SE [20], ARWEN [23] and manual annotation based on the anticodons and secondary structures, they reported the full set of tRNA genes in four other oribatid mite species: *Oribatula* sp*.*, *Platynothrus peltifer*, *Hermannia gibba* and *Nothrus palustris* [24]. The loss of tRNA genes appears to follow no specific rule, and there is no consensus on whether mt tRNA genes are really missing in oribatid mites.

In this study, we sequenced the complete mitogenome of one oribatid mite (Oribatulidae: *Oribatula sakamorii*). We also re-annotated the mitochondrial (mt) tRNA genes in all available oribatid mites. The following were our aims: (1) investigate whether tRNA genes are really lost in oribatid mites, (2) improve the accuracy of atypical tRNA annotation and document mt gene rearrangements in the oribatid mites investigated to date, (3) assess whether mt gene rearrangements hold promise as evolutionary and phylogenetic markers in oribatid mites and (4) explore the phylogenetic relationships of oribatid mites using complete mitogenomes.

## Methods

### Mite collection and DNA extraction

*O*. *sakamorii* specimens were collected from soil samples of a park in Wuhu, Anhui Province, China, on October 6, 2019, and preserved in alcohol. The mite samples were preserved at − 20 °C before DNA extraction. The mites were identified as *O*. *sakamorii* based on their morphology. To confirm identification, molecular techniques were applied. We used ClustalX2.0 [25] to compare the region of mt DNA sequences containing 18S rRNA genes to those in previously identified *O*. *sakamorii* isolates (GenBank accession number AB818530.1). All specimens were deposited at Wannan Medical College. Approximately 1000 adult mites were collected, and total genomic DNA was extracted using the phenol–chloroform method [26].

### Mitochondrial genome sequencing, assembly

One microgram of purified total genomic DNA of the holotype was fragmented and used to construct a paired-end library (insert size 300–500 bp) using the TruSeq^TM^Nano DNA Sample Prep Kit (Illumina, USA). The library was sequenced using the Illumina HiSeq 4000 platform (2 × 150 bp paired-end reads) at BIOZERON Co., Ltd. (Shanghai, China) [27]. The sequencing coverage of *Oribatula sakamorii* is 460×.

Prior to assembly, the raw reads were filtered to remove the reads with adaptors, those with quality score (Q) < 20, those containing a percentage of uncalled bases (“N” characters) ≥ 10% and duplicated sequences. The mitogenome was then reconstructed using a combination of de novo and reference-guided assemblies, and the following three steps were followed to assemble them. First, the filtered reads were assembled into contigs using SOAPdenovo 2.04 [28]. Second, the contigs were aligned to the reference mitogenome of *Oribatula* sp. (MH921998) using BLAST, and the aligned contigs (≥ 80% similarity and query coverage) were ordered according to the reference genome. Third, the clean reads were mapped to the assembled draft mitogenome to correct the incorrect bases, and the majority of gaps were filled through local assembly.

### Mitochondrial genome annotation and analysis

PCGs were identified using MITOS2 (http://mitos2.bioinf.uni-leipzig.de) and MEGA software [29]. The two rRNA genes, *rrnL* and *rrnS*, were identified in BLASTn searches (NCBI) based on highly conserved sequence motifs. However, the 5′- and 3′-end sequences of *rrnL* and *rrnS* could not be accurately determined. We tentatively annotated the 5′-ends of these genes immediately following the 3′-ends of the upstream gene and the 3′-ends of the rRNAs immediately preceding the downstream gene, with no gaps in between [24]. Different prediction methods have varying predictive potential. Owing to the atypical secondary structure of tRNAs, we used the available tRNA prediction software, tRNAscan-SE [20], ARWEN [23], MITOS [30] and MITOS2 (http://mitos2.bioinf.uni-leipzig.de), along with manual annotation. Our aim was to maximize the accuracy of gene annotation. Manual annotation is based on the comparison of nucleotide sequences in conserved regions (anticodon loop and anticodon arm) and/or sequence alignment with the homologs in related species. The MFE values were calculated for these structures (constrained analysis) using RNAfold [31]. The tRNA secondary structure with the lowest constrained MFE value was considered to be most likely [17].

The nucleotide composition, codon usage and RSCU were determined using MEGA. To calculate skewness, we used the following formula: AT skew = (A − T)/(A + T); GC skew = (G–C)/(G + C) [32].

### Gene rearrangement analyses and phylogenetic analysis

CREx was used to conduct a common interval analysis [33]. Pairwise comparisons using CREx were performed for all available oribatid mites and the major mt genes (PCGs and rRNAs) of the presumed ancestral arthropod *L*. *polyphemus* to determine the minimum number of genome rearrangement events separating each of these mite species from the “recently ancestral” state. CREx considers three types of rearrangement events: transpositions, reverse transpositions and reversal. We mapped the gene boundaries on the gene order to identify the unique, derived and ancestral gene boundaries between the ancestral arthropod *L*. *polyphemus* and other taxa of oribatid mites [34].

The complete mitogenome sequences of 57 species of Arachnida and 2 species of Xiphosura were retrieved from GenBank (Additional file 1: Table S1). The outgroup included 13 species belonging to 12 different orders: Xiphosura, Amblypygi, Araneae, Opiliones, Pseudoscorpiones, Ricinulei, Scorpiones, Solifugae, Thelyphonida, Holothyrida, Ixodida and Mesostigmata. The nucleotide sequences of the PCGs were aligned individually using Mafft v7v.7.035 [35] with a codon and protein strategy. Large gaps and ambiguous sites were omitted using Gblocks v.0.91b [36] with the G-INS-i strategy for global homology and were manually inspected before concatenation. The third codon of the aligned nucleotide sequences was additionally removed using MEGA6 [29] to eliminate the random or swinging sites. Three datasets were separately concatenated from the alignments of individual genes (13 whole-codoning nucleotide sequences, 13 excluded-third-codoning nucleotide sequences and 13 amino acid sequences) in Geneious v5.4 [37]. Phylogenetic analyses were performed using ML [38] and BI [39] methods.

Dataset partitioning was performed using PartitionFinder, based on an initial total of 39 data blocks (nucleotide sequences: 13 PCGs by three codon positions and 13 PCGs by the first and second codon positions; amino acid sequences: 13 PCGs). Each type of partitioned nucleotide sequence was independently run twice. The models were predicted using PartitionFinder v 0.2.1.1 [40] based on the Bayesian information criterion. PartitionFinder used unlinked branch lengths, a greedy search algorithm for nucleotide sequences and RAxML models. For the dataset of whole-codoning nucleotide sequences, the substitution model GTR + I + G was selected by PartitionFinder as the best model for 14 of the 20 partitions, whereas GTR + G models were selected for the remaining 6 partitions (Additional file 2: Table S2). For the dataset of excluded-third-codoning nucleotide sequences, the best substitution model GTR + I + G was selected by PartitionFinder for all eight partitions (Additional file 2: Table S2). For the amino acid sequence dataset, the substitution model LG + I + G was selected by PartitionFinder as the best for five of the seven partitions, and the LG + G models were selected for the remaining two partitions (Additional file 2: Table S2).

BI analyses were performed using MrBayes v.3.2.2 [39]. For the two datasets of nucleotide sequences, we used separate data partition models and performed two independent runs, each with four Markov chain Monte Carlo (one cold chain and three heated chains). Each of the two datasets was run for 20 million generations, with trees sampled every 1000 generations. The convergence of parameter estimates was performed using TRACER v.1.6. A conservative burn-in of 25% was applied. All estimated parameters showed ESS values > 200. For the amino acid sequence datasets, BI analyses were performed using MrBayes according to the same process, but a fixed (wag) model was used, besides running 10 million generations. The consensus tree, supported by BPP ≥ 95%, was edited using FigTree v.1.4.0. The nodes were considered strongly supported [41].

ML analyses were performed using RAxML-7.035 with the GTRGAMMAI model for nucleotide sequences and the PROTGAMMAWAG model for amino acid sequences. Clade support was assessed using nonparametric bootstrap with 1000 replicates. The consensus tree, supported by BSP ≥ 70%, was edited using FigTree v.1.4.0. The nodes were considered strongly supported [42].

## Results

### General features of *O*.* sakamorii* mitogenomes

The mitogenome of *O*. *sakamorii* (GenBank: MT232643) had an overall size of 14,494 bp. It was circular, with 13 PCGs, two ribosomal RNA (rRNA) genes and 22 tRNA genes and a large non-coding region (Additional file 3: Table S3). A software search and visual inspection of *O*. *sakamorii* sequences helped detect all tRNAs. We detected 21 reliable tRNAs using online tools, including tRNAscan-SE [20], ARWEN [23], MITOS [30] and MITOS2 (http://mitos2.bioinf.uni-leipzig.de). *trnV* was detected manually by alignment with homologous species based on the anticodons and secondary structures (Table 1). *trnF*, *trnW*, *trnE* and *trnK* formed typical cloverleaf structures. The other 18 tRNAs had reduced D- and T-arms or both arms.

**Table 1:** Alignment of the nucleotide sequences of four mitochondrial tRNA genes (*trnV*, *trnM*, *trnG* and *trnY*) in the same superfamily in oribatid mites

*trnV*	*Oribatula sakamorii*	TTAGGGT	-TTA-TTTTT	CTTTAAT**TAC**GGTAAAG	A-TG-TTTTT	-AACC-TTAG
	*Oribatula* sp.	T-GTTGGG	GCTTTTCTTTTGAGAT	TTTTATT**TAC**GGTAAAA	ATTTG-TCTGC	CCTAAC-TT
	*Paraleius leontonychus*	AGA-GTTT	-TGG-TTCC	CTTTAAT**TAC**GGTAAAG	GT-TGGTGT	-TAACTCTT
*trnM*	*O. sakamorii*	-AGCAAGT	-AAAGCTTAATT-AAGCTT	AGGTATTCATAATTCCT	AGAATTT	CATTGCTA
	*Oribatula* sp.	T-AGTAAA	C AAAGCTTAACCACAAAGCTT	AGGTATT**CAT**AATTCCT	AGAAATTGA	A-TTACTAG
	*Pa. leontonychus*	-AGCAACT	-AAAGCTTAAATA-AAGCTT	AGGTATT**CAT**AATTCCT	AGAACAAC	CGTTGCTC
*trnG*	*O. sakamorii*	AT-TCTTA	TAG-TATATGTC-GT-ACA	-TTTAATT**TCC**AATTAAA	AAG-AAA	AAAGAAAA
	*Oribatula* sp.	ATTTCA-A	CAG-TATACAAA-GT-ACG	C-TTAATT**TCC**AATTAAG	AAG-AAAA	AA-GAAACA
	*Pa. leontonychus*	AA-TCTTA	AAAATTAAAAGGTAAAGTTAAAT	-TAAAATT**TCC**sCTGT	GAGTCACAGC	TTT GATAC
*trnY*	*O. sakamorii*	TTTGA-GG	TGATTACGCAT-A	AGAAA-TT**GTA**AA-TTTCT	TTTTTT–– T	TA-TCAAAT
	*Oribatula* sp.	TTT-ACGG	TGA-GATTGGA	AGAAA-TT**GTA**AA-TTTCT	ATTTTTTTAT	CA-TTATAT
	*Pa. leontonychus*	TTT-AGGG	TGAAAA-GCAAA-A	-CAAAGTT**GTA**AACTTTG	-TTTGCAAAT	CGGTGTAA

The anticodons are boldfaced, and the conserved sequences are underlined

**Fig. 1:**
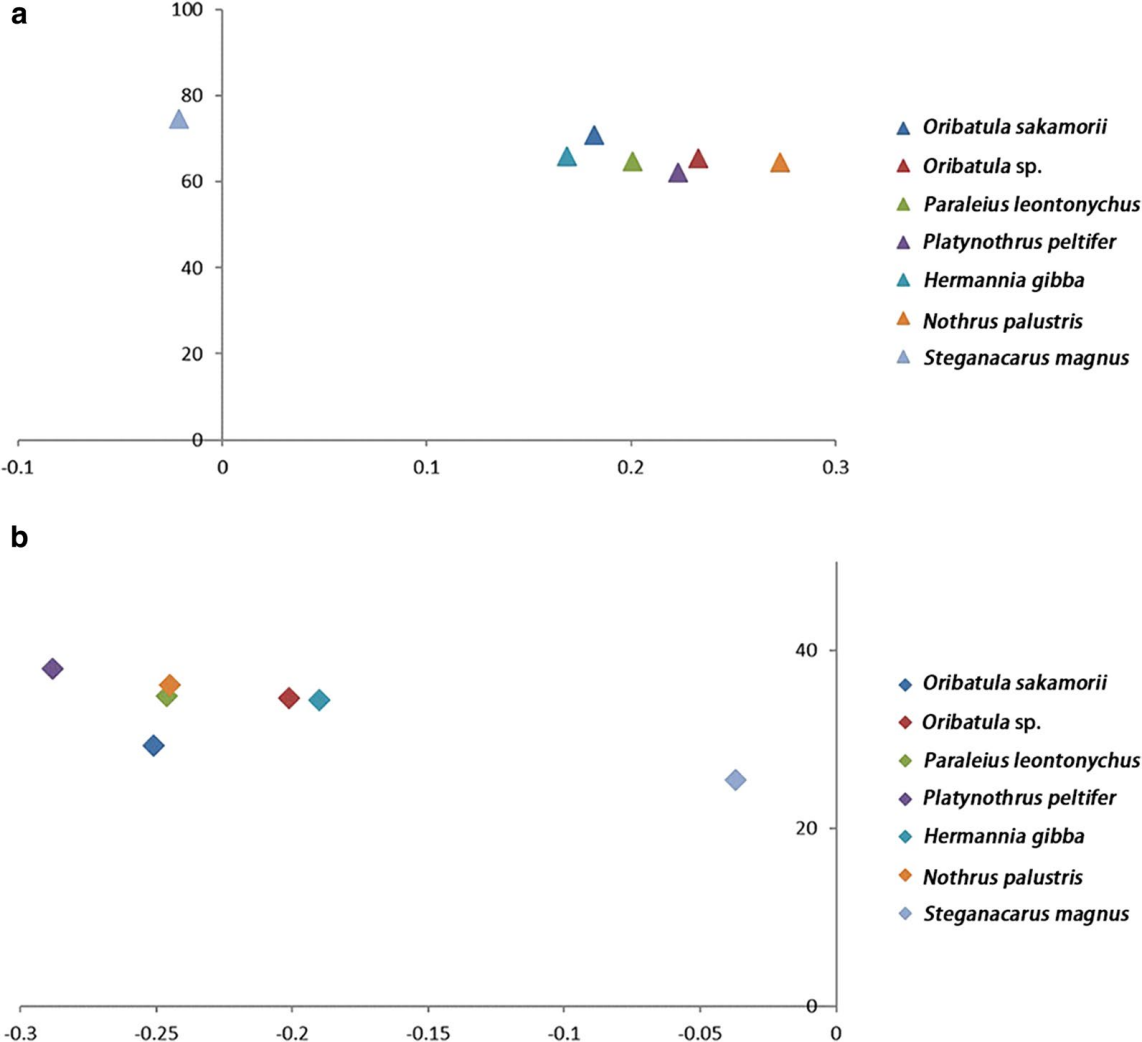
**a** AT% *vs* AT-skew and **b** GC% *vs* GC-skew. Values are calculated on the (−)-strands for full-length mitochondrial genomes. The X-axis indicates the level of nucleotide skew, and the Y-axis indicates the nucleotide percentages

The percentage nucleotide composition of the mt (+)-strand was A = 41.7, C = 18.2, G = 10.9 and T = 29.1, which resulted in a positive AT-skew (0.182) and a negative GC-skew (-0.251). A comparative analysis of the A + T% *vs* AT-skew and G + C% *vs* GC-skew among the available mt-genomes of oribatid mites indicated the clubbing of six oribatid mites, with only *S*. *magnus* separated from this group (Fig. 1). This is consistent with the differences observed in the supercohort of oribatid mites (Additional file 1: Table S1).

### Re-annotation of mt tRNA genes in oribatid mites

**Fig. 2:**
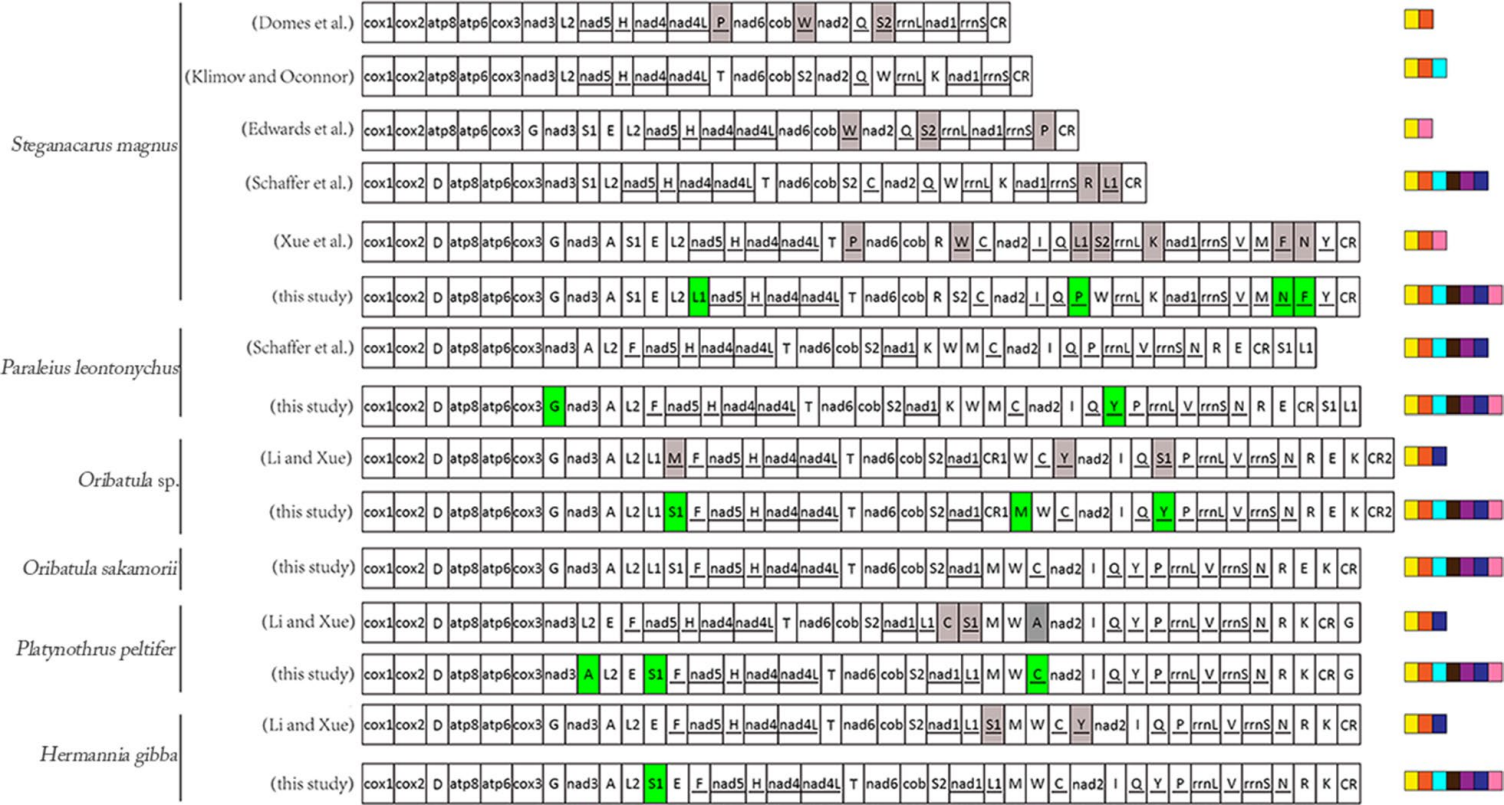
Mitochondrial gene orders of the six oribatid mite species. The mitogenome orders obtained from different annotations for *Steganacarus magnus*, *Paraleius leontonychus*, *Oribatula* sp*.*, *Hermannia gibba* and *Platynothrus peltifer.* The gray boxes indicate re-annotated genes. The green boxes indicate the newly predicted tRNAs. The underlined genes were present on the (−)-strand. The genes are presented in the original order. Intergenic distances are not included, and sizes of genes are not to scale. The tRNA annotation methods are indicted in different colors at the end of each sequence (tRANscan-SE in yellow, ARWEN in orange, minimum free energy in light blue, MITOS in black, MITOS2 in purple, manual annotation using anticodon and secondary structure in blue and manual annotation using sequence alignmentsin pink)

**Fig. 3:**
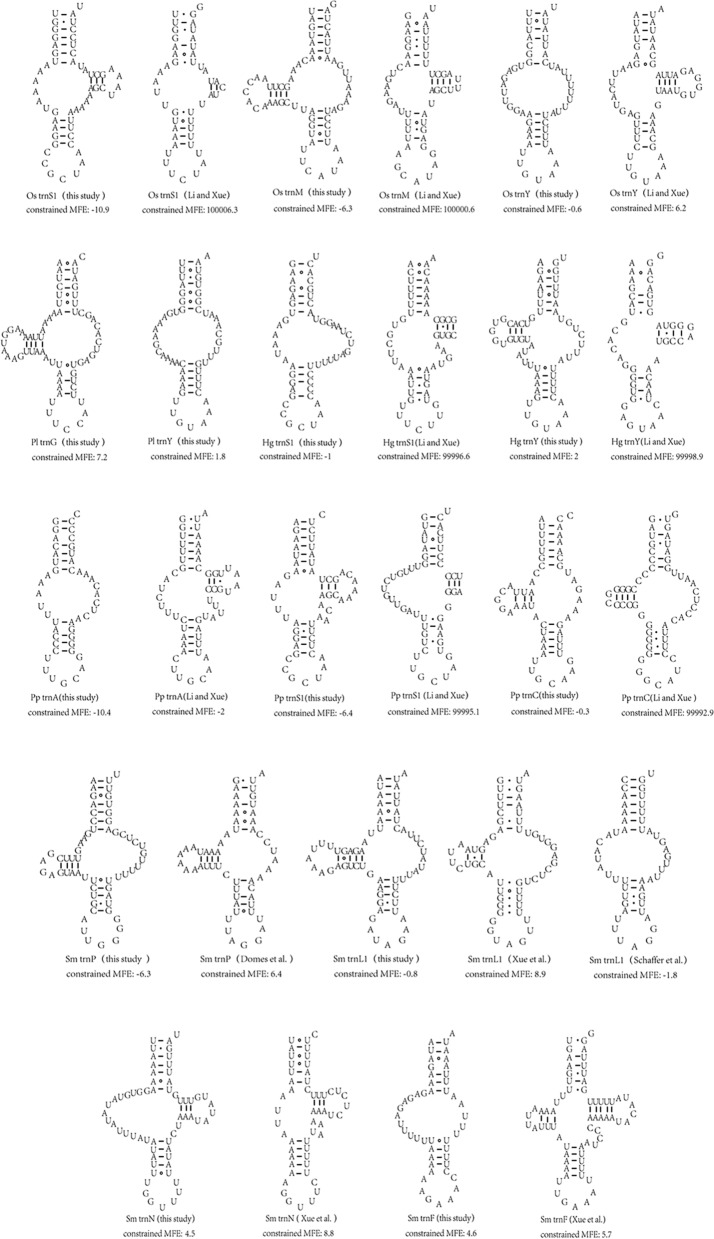
Comparison of the secondary structures of tRNAs. Two *Paraleius leontonychus* (Pl) tRNAs were retrieved. MFE: minimum free energy

Different methods have varying predictive potential for the atypical secondary structure of tRNAs. For example, the tRNA annotation results for the *S*. *magnus* mt sequence differ when different types and/or numbers of tRNA predictive methods are used (Fig. 2). In the present study, we applied all available tRNA prediction methods designed to maximize the accuracy of tRNA gene annotation, particularly for atypical tRNAs. We confirmed the prediction of the majority of tRNAs using previously described methods. All the “lost” tRNA genes in oribatid mites, along with some of the incorrectly annotated tRNAs, were re-annotated (Fig. 2). The details are as follows:

The re-annotation of the tRNAs of *Oribatula* sp. indicated the presence of three tRNAs based on its mitogenome (GeneBank: MH921998), namely *trnY*, *trnS1* and *trnM* [24]. ARWEN [23] predicted that *trnY* is located between *trnQ* and *trnP*, whereas the previously retrieved *trnY* is located between *trnC* and *nad2* (Fig. 2). *trnS1* was detected using MITOS2, encoded on the (+)-strand at the 3′-end of *trnL1*. In contrast, the previously described *trnS1* was detected on the (−)-strand at the 3′-end of *trnQ* (Fig. 2). *trnM* could be identified manually by sequence alignment and secondary structure comparison with sequences identified in other species of oribatid mites (Table 1). To select the most likely tRNA sequences, the MFE values were calculated [31]. The MFE values obtained for this study indicated three tRNA structures that were smaller than those observed in a previous study (Fig. 3). The secondary structure of the tRNAs had fewer mismatches in the acceptor and/or anticodon stem (Fig. 3).

Using multiple sequence alignment analysis (Table 1), we manually identified two “lost” tRNAs (*trnG* and *trnY*) in *Pa*. *leontonychus* [17]. *trnG* was detected in the (+)-strand at the 3′-end of cox3. *trnY* was detected on the (−)-strand at the 3′-end of *trnQ* (Fig. 2). The MFE values and secondary structures of *trnG* and *trnY* are presented in Fig. 3. Using manual sequence annotation, we also re-annotated *trnA*, *trnS1* and *trnC* for *Pl*. *peltifer* and *trnS1* and *trnY* for *H*. *gibba* (Fig. 2). The MFEs for these tRNA structures were lower than those described by Xue et al. [24] (Fig. 3). The secondary structures of the tRNAs described by us also showed fewer mismatches in the acceptor and/or anticodon stem (Fig. 3).

The mitogenome annotation of *S*. *magnus* has been controversial. In this study, we confirmed the predictions for 18 tRNAs reported in previous studies, and four tRNAs (*trnP*, *trnL1*, *trnN* and *trnF*) were re-annotated (Fig. 2). The position of *trnP* remains undetermined. In their version of annotation, Domes et al. predicted that *trnP* was located between *nad4L* and *nad6*, and the same was confirmed by Xue et al. However, Klimov and Oconnor, Schäffer et al. and Edwards et al. refuted the *trnP* annotation predicted by Domes et al. Additionally, Edwards et al. re-annotated *trnP* [17–19, 21, 22]. Based on manual annotation, *trnP* was found to be located in the sequence of *trnQ* ~ *trnW* [on the (−)-strand] (Fig. 2). Compared to previous studies describing *trnP*, the structure of *trnP* in this study was found to have greater thermodynamic stability (Fig. 3). The secondary structure of *trnL1* predicted by Xue et al. showed five mismatches in the anticodon stem [22] (Fig. 3). The *trnL1* structure suggested by Schäffer et al. showed two mismatches in the anticodon stem [17]. We proposed that the *trnL1* structure has one mismatch in the anticodon stem, indicating that our predicted structure may have greater stability (Fig. 3). The *trnL1* structure predicted by us also conserved the *Limulus polyphemus* gene order *trnL2*-*trnL1* (Fig. 2). The *trnN* structure could be inferred manually in this study. The MFE values in our analyses were lower than those reported by Xue et al. [22]. This indicates that the *trnN* structure predicted by us may be more stable. Based on the anticodon and secondary structures, we manually confirmed that *trnF* is located between the *trnN* and *trnY* genes (Fig. 2), whereas Xue et al. described an overlap with the first position of an upstream gene (39 bp) [22], which may violate the tRNA punctuation model for RNA processing [43].

### Codon usage in the mitogenomes of oribatid mites

**Fig. 4:**
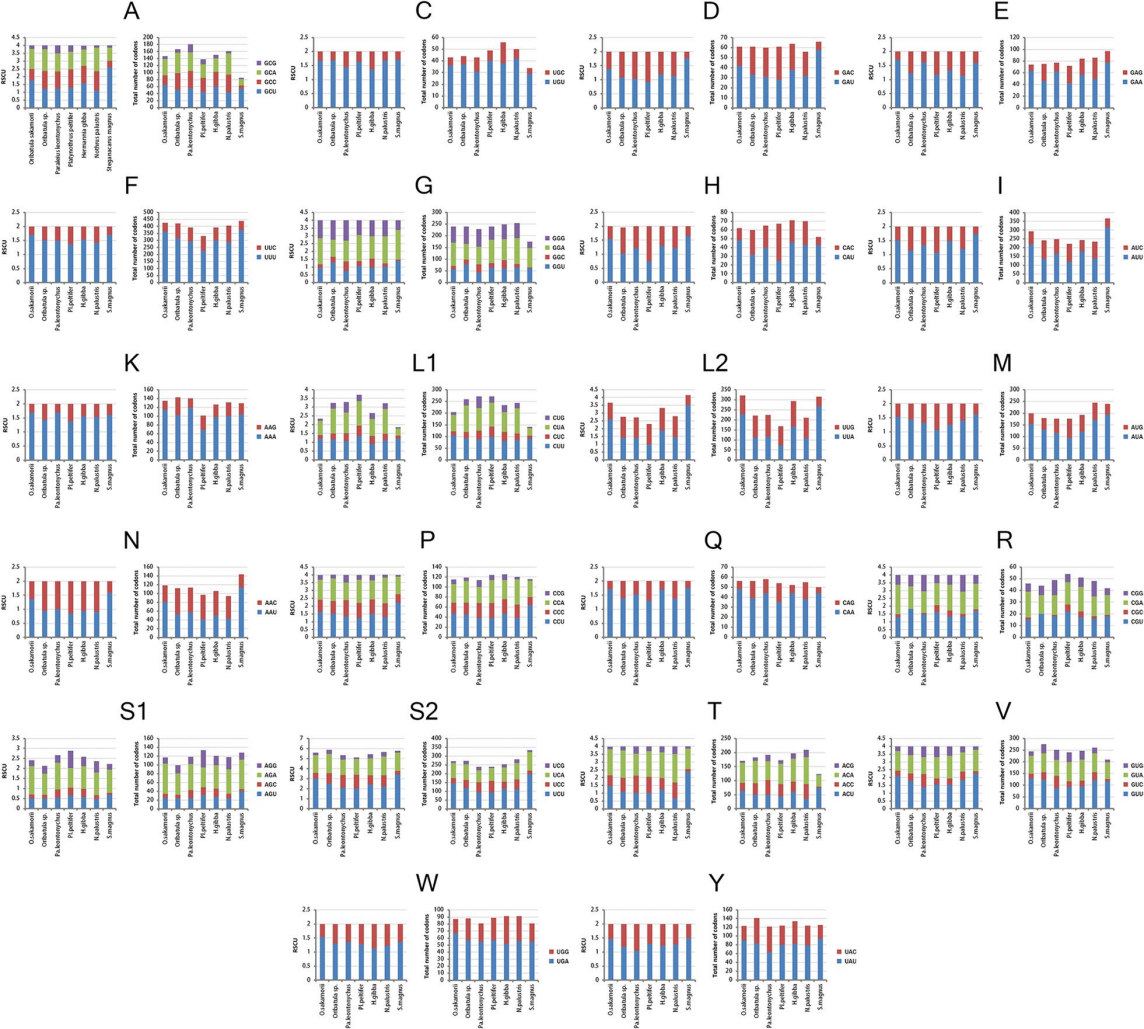
Relative synonymous codon usage (RSCU) and codon numbers of the 22 amino acids. The X-axis indicates the oribatid mite species; the Y-axis indicates the RSCU or total number of codons

We analyzed the codon usage in the mt PCGs of the seven species of oribatid mites to determine whether the corresponding codons of the “lost” tRNA genes were used. To avoid bias resulting from unusual putative start codons and incomplete stop codons, all the first codons and stop codons were excluded from the analysis. The codons for the 22 amino acids were present in all the PCGs from the oribatid mites, including the two species in which mt tRNA gene “lost” was reported (Fig. 4). The amino acid frequencies in the seven different oribatid mites were similar. The codon families also exhibited a similar pattern among them.

### Common interval analysis of Sarcoptiformes gene order using CREx

**Fig. 5:**
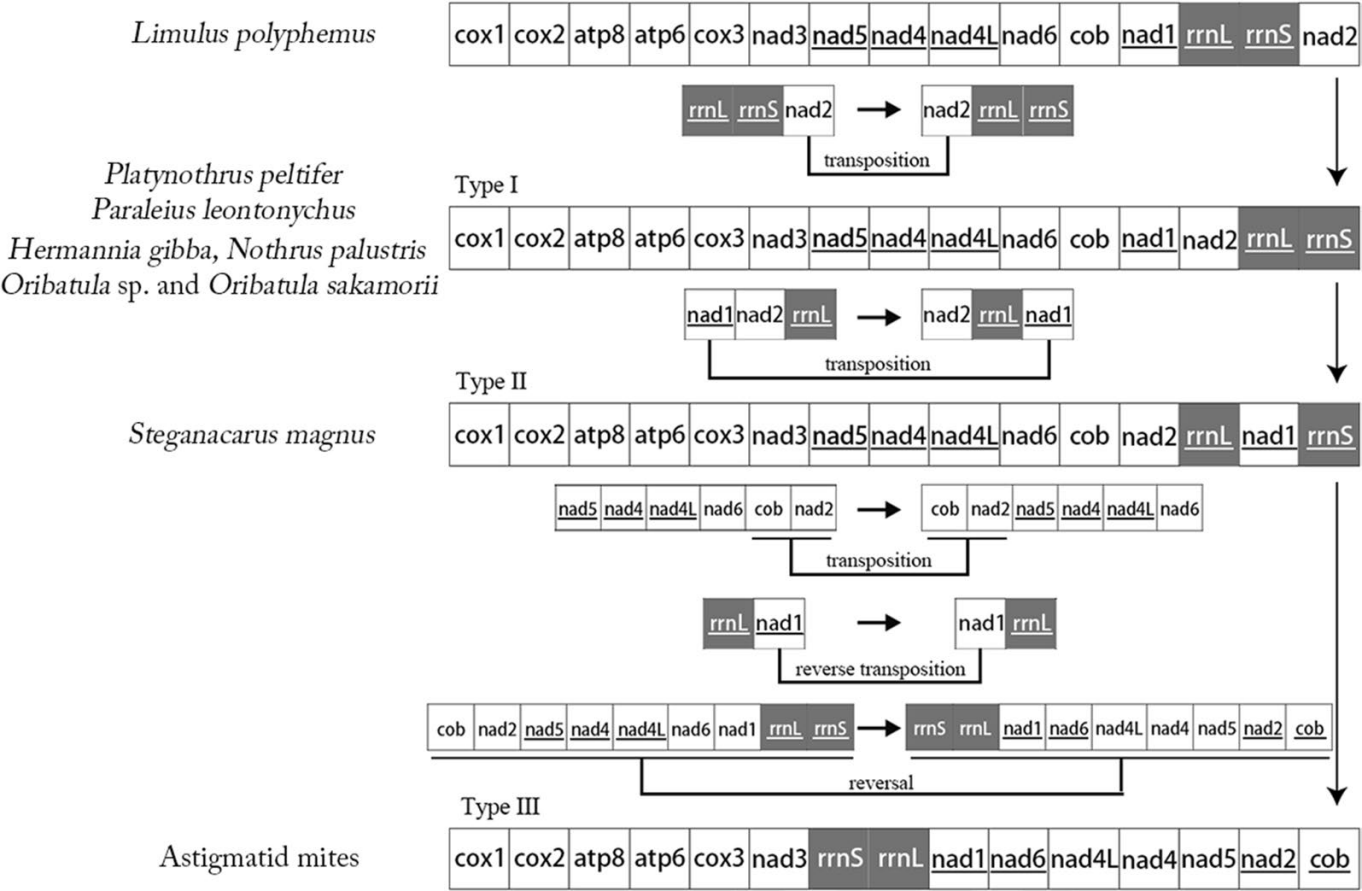
Evolution of gene orders [protein-coding genes (PCGs) and rRNAs] in mitogenomes explained using CREx. Rearrangement operations occurred from an inferred ancestral arthropod gene order to oribatid mites followed by astigmatid mites. Type I indicates the mt PCG and rRNA gene orders in six oribatid mites. Type II indicates the mt PCG and rRNA gene order in *Steganacarus magnus*. Type III indicates the mt PCG and rRNA gene orders in astigmatid mites. Underlined genes are present on the (−)-strand. The genes are presented in their original order; intergenic distances are not included, and the gene sizes are not true to scale. The *rrnL* and *rrnS* genes are color-coded (black gray in color)

Using CREx, we calculated the common interval as a measure of the extent of mt genome reorganization (Table 2). Based on the number of common intervals, the gene orders of the major genes (PCGs and rRNAs) were used to infer the possible evolutionary relationships within oribatid mites, astigmatid mites and the putative ancestral arthropod (*L*. *polyphemus*) (Fig. 5). By comparing the gene order of type I with *L*. *polyphemus*, *nad2* was indicated as a transposition. Comparing of the gene order of type II with type I indicated *nad1* as a transposition. By comparing the gene order of type III with type II, three rearrangement events were inferred: transposition of one gene block (*cob* and *nad2*), reverse transposition of two genes (*nad1* and *rrnL*) and four reversals in a large gene block (Fig. 5).

**Table 2:** Number of common intervals of *Limulus polyphemus*, oribatid mites and astigmatid mites detected upon comparison of the major mitochondrial gene [protein-coding genes (PCGs) and rRNAs] arrangements are compared

	*L*. *polyphemus*	Six oribatid mites	*S*. *magnus*	Astigmatid mites
*Limulus polyphemus*	204	154	132	56
Six oribatid mites	154	204	154	56
*Steganacarus magnus*	132	154	204	60
Astigmatid mites	56	56	60	204

Six oribatid mites, including *Hermannia gibba*, *Nothrus palustris*, *Oribatula* sp., *Oribatula sakamorii, Platynothru Peltifer* and *Paraleius leontonychus*, shared the same gene (PCGs and rRNAs) order. All available astigmatid mites shared the same gene (PCGs and rRNAs) order

### Mapping of shared gene orders in oribatid mites

**Fig. 6:**
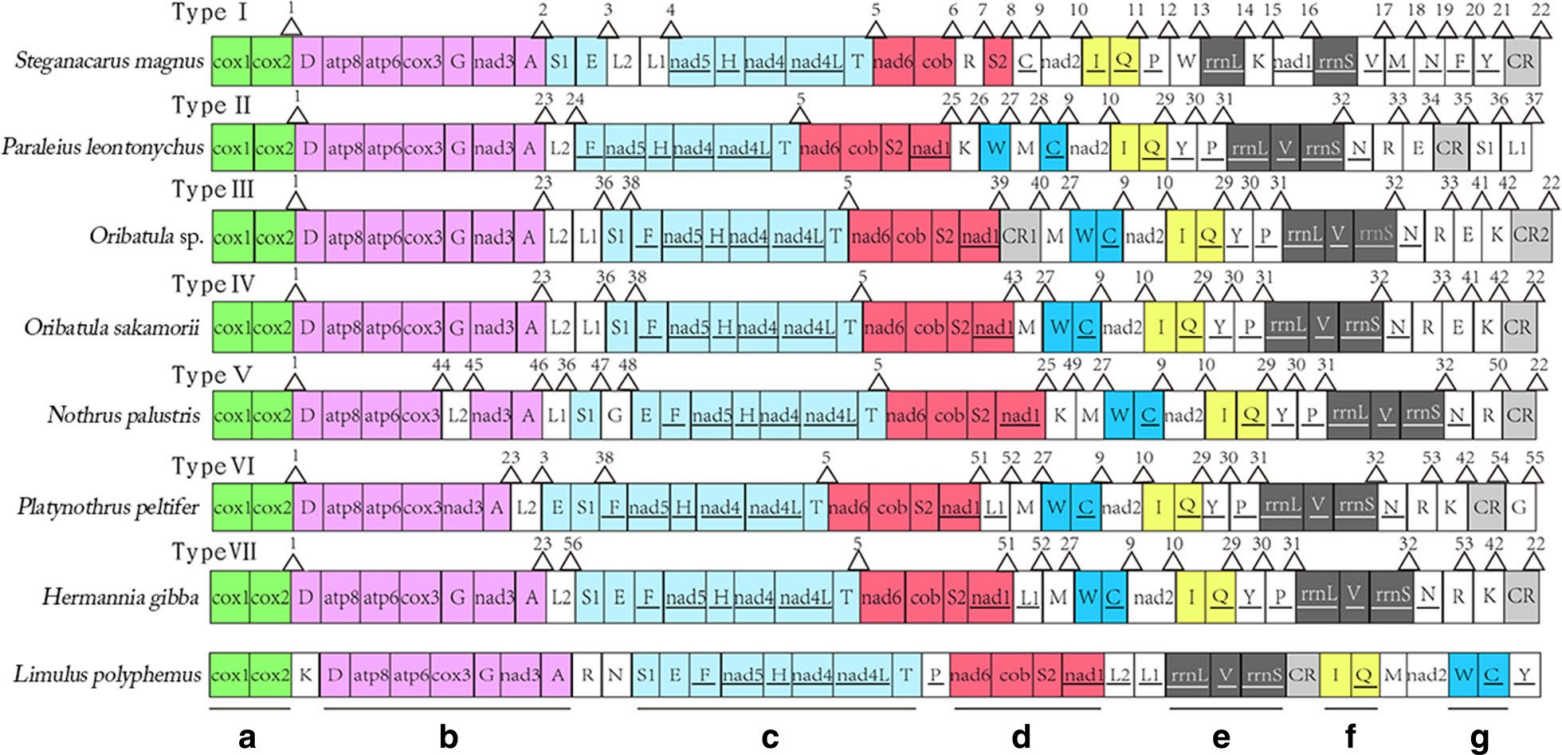
Gene order representation. Underlined genes were present on the (−)-strand. The ancestral gene blocks a–g are underlined in the *Limulus polyphemus* gene order and also indicated by different colors. Different codes were used to label the boundaries

The linearized gene order maps for seven oribatid mite mitogenomes and the ancestral arthropod gene order are shown in Fig. 6. The mitogenome of oribatid mites was rearranged in comparison to that of the hypothetical ancestor of arthropods, represented by *L*. *polyphemus*. Seven oribatid mite species from six families showed rearranged mitogenomes. Each species had a unique mitogenome organization (Type I to Type VII, Fig. 6). Seven ancestral gene blocks consisted of two to eight genes (1–7 gene boundaries) (Fig. 6). Gene block a (*cox1*-*cox2*) and block f (*trnI*-*trnQ*) were conserved in all seven oribatid mites. Gene block b (*trnD*-*atp8*-*atp6*-*cox3*-*trnG*-*nad3*-*trnA*) was retained in all oribatid mite species, except in *Pl*. *peltifer* and *N*. *palustris*. Block c (*trnS1*-*trnE-trnF*-*nad5*-*trnH*-*nad4*-*nad4L*-*trnT*) was only conserved in *H*. *gibba* and *Pl*. *peltifer* and partially present in the other five oribatid mites. Gene block d (*nad6*-*cob*-*trnS2*-*nad1*) was retained in all oribatid mite species, except *S*. *magnus*, in which *trnR* was inserted between *cob* and *trnS2*, and *nad1* appeared as a translocation. Block f (*rrnL*-*trnV*-*rrnS*) and block g (*trnW*-*trnC*) were also conserved in all oribatid mite species, except in *S*. *magnus*.

One hundred twenty-nine derived gene boundaries were detected in the seven available mitogenomes of oribatid mites (numbered 1 to 56 in Fig. 6). Of these, 19 were shared, derived gene boundaries present in at least two species. An additional 37 unique boundaries found in a single species were identified (Fig. 6). Certain gene boundaries (2, 4, 6–8 and 11–21) were detected only in *S*. *magnus*. Gene boundaries (24–26, 28, 34, 35 and 37) were found only in *Pa*. *leontonychus*. Gene boundaries 39 and 40 were detected only in *Oribatula* sp*.* Gene boundary 43 was detected only in *O*. *sakamorii*. Gene boundaries (43–50) were detected only in *N*. *palustris*. Gene boundaries 54 and 55 were detected only in *Pl*. *peltifer*. Gene boundary 56 was detected only in *H*. *gibba*.

**Fig. 7:**
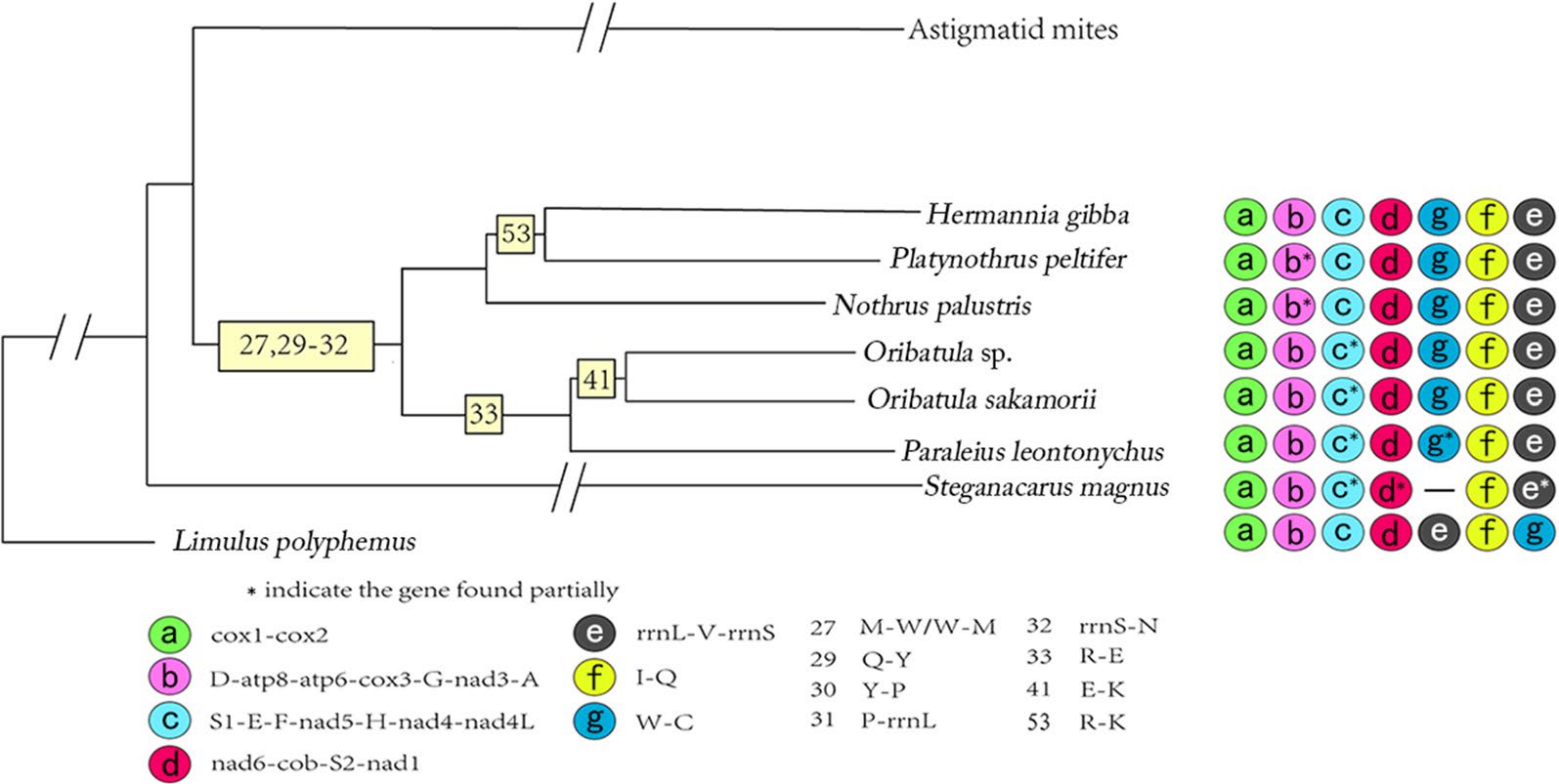
Representation of derived characters on a phylogenetic tree. A part of Bayesian inference is used for the representation of the ancestral and shared derived characters. The shared derived character states are shown on the node. The ancestral gene blocks (a–g) are indicated using different colors and codes, which are shown at the terminal end of the branch. Partial ancestral characters are marked with an asterisk

Of the 19 derived gene boundaries, 11 were either homoplastic or secondarily lost in some of the taxa descending from the node, and the remaining 8 were unambiguous synapomorphic. Hence, eight shared derived gene boundaries were mapped onto the phylogenetic tree inferred from the maximum likelihood (ML) dataset (Fig. 7). Five derived gene boundaries (27 and 29–32) were synapomorphies in the supercohort Desmonomatides (Type II to Type VII, Fig. 6). Gene boundary 41 (*trnE*-*trnK*) was a synapomorphy for the family Oribatulidae (Type III and Type IV, Fig. 6). Gene boundary 33 (*trnR*-*trnE*) was a synapomorphy for the superfamily Oripodoidea (Type II to Type IV, Fig. 6). Meanwhile, boundary 53 (*trnR*-*trnK*) was a synapomorphy for *H*. *gibba* and *Pl*. *peltifer* (Type VI to Type VII, Fig. 6).

### Phylogenetic relationships

**Fig. 8:**
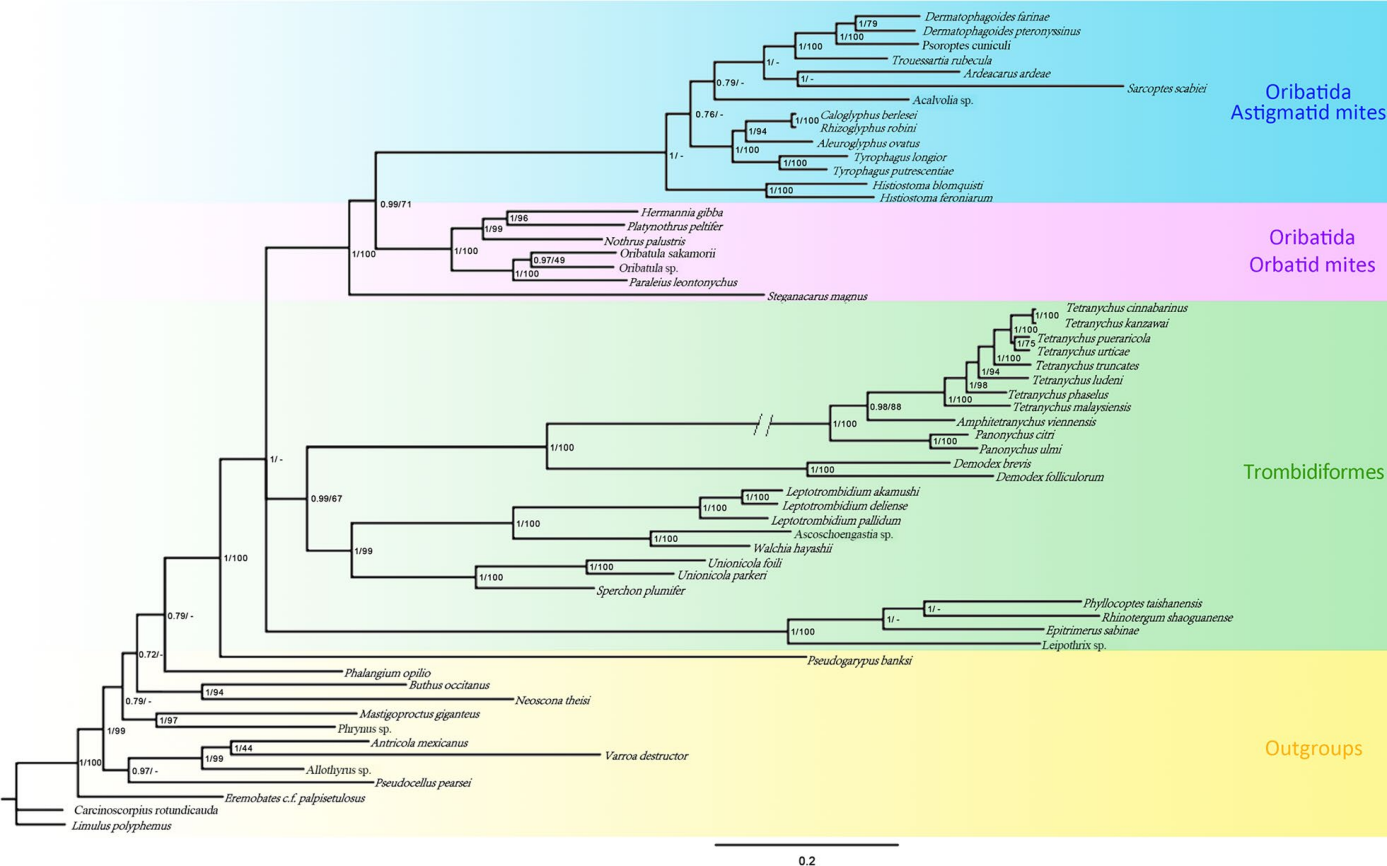
Phylogenetic tree inferred from mitochondrial genome sequences using maximum likelihood and Bayesian inference methods. The branch lengths presented here follow the Bayesian analysis. The node numbers indicate Bayesian posterior probabilities (BPP) and maximum likelihood bootstrap proportion (BSP). “−” indicates the absence of the node in the corresponding analysis. The numbers indicate BPP and BSP values from the analyses of datasets consisting of without third codon positions of protein coding genes

We constructed a phylogenetic tree based on a nucleotide dataset from the 13 mt PCGs of 59 mites (Fig. 8). ML and Bayesian inference (BI) analyses revealed similar topologies. The monophyly of Oribatida was recovered with strong support [Bayesian posterior probabilities (BPP) = 1, bootstrap proportion (BSP) = 100] (Fig. 8), alongside the monophyly of Desmonomatides. The monophyly of Astigmata (astigmatid mites) was recovered with support (BPP = 0.99, BSP = 71). Within Desmonomatides, six oribatid mites from two superfamilies (Crotonioidea and Oripodoidea) of two cohorts (Nothrina and Brachypylina) formed a monophyletic clade; each superfamily or cohort was monophyletic with strong support (BPP = 1, BSP = 100). Here, the phylogeny of Desmonomatides at the cohort level was suggested as [Astigmata, (Nothrina, Brachypylina)]. The novel sequence of *O*. *sakamorii* exhibited a sister group relationship with the *Oribatula* sp. sequence (BPP = 0.97, BSP = 49) (Fig. 8). The phylogenetic tree indicated that *H*. *gibba* and *Pl*. *peltifer* clustered into one branch with strong support (BPP = 1, BSP = 96) (Fig. 8).

## Discussion

The tRNA genes present in mitogenomes are critical for the mt translation system. The loss of any of the 22 tRNA genes severely affects the mt translation system unless a nuclear equivalent is imported into the mitochondria. Based on this, Xue et al. did not support the loss of mt tRNA genes in Sarcoptiform mites [22]. Additionally, Fang et al. did not support tRNA loss. After de novo sequencing and analysis of the circular mitogenome of *Tyrophagus putrescentiae*, the authors retrieved three tRNA (*trnF*, *trnS1* and *trnQ*) genes that previous studies had indicated as “lost” [44]. We identified all “lost” tRNAs in oribatid mites. In addition, the overall codon usage was found to be considerably similar across the available oribatid mite sequences. Therefore, our results do not support the loss of any of the 22 tRNA genes in oribatid mites.

Accurate annotation is necessary to determine the degree of rearrangement within a species. However, the identification of tRNAs lacking one or both arms or containing mismatches in the stems is challenging [17]. In addition, different annotation methods can influence the results of sequence annotation [45]. Improvement in the accuracy of gene annotation will benefit the downstream users of these gene sequences. We corrected the incorrect annotation of atypical tRNAs in the reported oribatid mite sequences using a multi-software combined manual annotation approach.

The relationship between oribatid mites and astigmatid mites is controversial. The relationship based on major genes has not been evaluated in earlier studies. The rearranged mt gene order will help clarify the relationship between oribatid mites and astigmatid mites. Limiting our CREx analysis to only major genes (PCGs and rRNAs) and removing tRNAs improved our understanding of the major genomic evolutionary events within the Metazoa. This is because the higher relative rates of rearrangement obscured the fundamentally conserved nature of gene arrangement across taxa [30]. Therefore, using the major genes, we mapped possible evolutionary processes within Sarcoptiformes mites (Fig. 6). Our CREx results are congruent with previous hypotheses, including those derived using morphological [46] and molecular approaches [8], as well as the fossil record that validates the origin of astigmatids from oribatid mites [47, 48]. However, further studies that consider gene order variations in additional mitogenomes are necessary.

Oribatid mite mitogenomes are characterized by low rates of gene rearrangement, with six or seven gene blocks conserved between any oribatid mite species and the ancestral arthropod mitogenome. When the relative order of the major genes (PCGs and rRNAs) was considered, only one or two genes were found to be rearranged relative to their position in the ancestral genome. However, the ancestral mt gene order features in oribatid mites have not been determined because the taxa representing the basal species of this suborder have not been identified [6]. However, the conserved gene block e [(−) *rrnL*- (−) *trnV*- (−) *rrnS*], which shows preservation of the *L*. *polyphemus* gene order, has been detected in six oribatid mites. Therefore, the hypothetical ancestor of Acariformes mites might also retain this conserved gene block. This finding amends the hypothesis proposed by Xue et al. on the ancestral gene order of Acariformes [49].

By mapping the derived gene boundaries, we identified several boundaries that were synapomorphic for major clades within oribatid mites, which supported the consensus phylogenetic topology. For example, *trnR*-*trnK* (derived boundary #53) was found to be synapomorphic for *H*. *gibba* and *Pl*. *peltifer*. The systematic position of *Hermannia* has been viewed differently in pervious morphological classifications [11, 12]. *trnE*-*trnK* (derived boundary #41) was found to be synapomorphic for Oribatulidae, which increased the confidence in clades with weak nodal support (Fig. 7).

The inference of “true” phylogenetic affinities and classifications within Acariformes was found to be challenging. The phylogenetic reconstruction performed by us based on 13 PCGs indicated that astigmatid mites are nested in oribatid mites. It is challenging to establish the correct systematic position of *Hermannia* in the Crotonioidea superfamily based solely on morphological observations [50]. The phylogenetic tree constructed by us showed that *H*. *gibba* and *Pl*. *peltifer* were clustered into one branch with strong support (BPP = 1, BSP = 96) (Fig. 4). Further mitogenome sequencing of oribatid mites from the Crotonioidea superfamily will be necessary to clarify the systematic position of *Hermannia*.

## Conclusions

We adopted a multi-software approach combined with a manual annotation to identify the mt tRNA genes previously reported as “lost” in oribatid mites. The tRNAs had unusual secondary structures and contained multiple nucleotide mismatches in their arms. The newly sequenced mitogenome of *O*. *sakamorii* has important ramifications for our understanding of tRNAs. The loss of tRNA genes is not universal in oribatid mites. We determined the correct systematic position of *Hermannia* and provided evidence supporting the fact that astigmatid mites are nested in the oribatid mite. The derived gene boundaries of oribatid mites serve as a valuable source of information for understanding oribatid mite phylogeny and evolution. However, extensive data on additional taxa of oribatid mites, including species belonging to each of the five supercohorts, could further enhance our knowledge of rearrangements and other evolutionary events.

## Supplementary Information

**Additional file 1**: **Table S1**. Mite species used in the present study (DOCX 23 KB)

**Additional file 2**: **Table S2**. Partition schemes used in the present study (DOCX 16 KB)

**Additional file 3**: **Table S3**. Mitochondrial genome organization of *Oribatula sakamorii* (DOCX 20 KB)

**Additional file 4**: **Figure S1**. Secondary structures of tRNAs of *Oribatula sakamorii* (Osa). The tRNA short name and the calculated constrained MFE is indicated (TIF 274 KB)

## Data Availability

The datasets generated and/or analyzed during the current study are not publicly available due (the mt genomes of *Oribatula sakamorii* submitted on GenBank under the accession number MT232643, and not released yet) but are available from the corresponding author on reasonable request.

## Abbreviations

*atp6* and *atp8*: Genes for ATP synthase subunits 6 and 8
bp: Base pair
cob: Gene for cytochrome b
*cox1*, *cox2* and *cox3*: Genes for cytochrome coxidase subunits 1, 2 and 3
CR: Control region
DNA: Deoxyribonucleic acid
RNA: Ribonucleic Acid
mt: Mitochondrial
*nad1* and *nad2*: Mitochondrial genes for NADH dehydrogenase subunits 1 and 2
PCGs: Protein-coding genes
PCR: Polymerase chain reaction
rRNA: Ribosomal RNA
*rrnS* and *rrnL*: Genes for small and large subunits of ribosomal RNA
RSCU: Relative Synonymous Codon Usage
*trnA*: TRNA gene for alanine
tRNA: Transfer RNA
*trnC*: TRNA gene for cysteine
*trnD*: TRNA gene for aspartic acid
*trnE*: TRNA gene for glutamic acid
*trnF*: TRNA gene for phenylalanine
*trnG*: TRNA gene for glycine
*trnH*: TRNA gene for histidine
*trnI*: TRNA gene for isoleucine
*trnK*: TRNA gene for lysine
*trnL1*: TRNA gene for leucine
*trnL2*: TRNA gene for leucine
*trnM*: TRNA gene for methionine
*trnN*: TRNA gene for asparagine
*trnP*: TRNA gene for proline
*trnQ*: TRNA gene for glutamine
*trnR*: TRNA gene for arginine
*trnS1*: TRNA gene for serine
*trnS2*: TRNA gene for serine
*trnT*: TRNA gene for threonine
*trnV*: TRNA gene for valine
*trnW*: TRNA gene for tryptophan
*trnY*: TRNA gene for tyrosine
MFE: The minimum free energy
ML: Phylogenetic analyses of maximum likelihood
BI: Bayesian inference
BPP: Bayesian posterior probabilities
BSP: Bootstrap proportion
