# Supplementary material for: Mitochondrial analysis of oribatid mites provides insights into their atypical tRNA annotation, genome rearrangement and evolution
Source: Parasit Vectors. 2021 Apr 23;14:221. doi: 10.1186/s13071-021-04719-0 (PMC8063316; doi:10.1186/s13071-021-04719-0)
Supplement: Supplementary file 1 — Additional file 1: Table S1. Mite species used in the present study (DOCX 23 KB) [file 13071_2021_4719_MOESM1_ESM.docx]

**Table S1.** Mite species used in the present study

| Order | Supercohort | Cohort | Superfamily | Family | Species | GenBank ID | Size(bp) |
| --- | --- | --- | --- | --- | --- | --- | --- |
| Sarcoptiformes | Desmonomatides | Astigmata | Analgoidea | Pyroglyphidae | *Dermatophagoides* *farinae* | NC_013184 | 14266 |
|  |  |  |  |  | *Dermatophagoides* *pteronyssinus* | EU884425 | 14203 |
|  |  |  |  | Trouessartiidae | *Trouessartia* *rubecula* | MH208456 | 14125 |
|  |  |  | Pterolichoidea | Pterolichidae | *Ardeacarus* *ardeae* | KY352304 | 14069 |
|  |  |  | Sarcoptoidea | Psoroptidae | *Psoroptes* *cuniculi* | KJ957822 | 14247 |
|  |  |  |  | Sarcoptidae | *Sarcoptes* *scabiei* | CM003133 | 13667 |
|  |  |  | Hemisarcoptoidea | Winterschmidtiidae | *Acalvolia* sp. | MH921997 | 14711 |
|  |  |  | Acaroidea | Acaridae | *Caloglyphus* *berlesei* | KF499016 | 14273 |
|  |  |  |  |  | *Rhizoglyphus* *robini* | MF596168 | 14244 |
|  |  |  |  |  | *Aleuroglyphus* *ovatus* | KC700022 | 14328 |
|  |  |  |  |  | *Tyrophagus* *longior* | KR869095 | 13271 |
|  |  |  |  |  | *Tyrophagus* *putrescentiae* | MK_013184 | 14156 |
|  |  |  | Histiostomatoidea | Histiostomatidae | *Histiostoma* *blomquisti* | KX452726 | 15892 |
|  |  |  |  |  | *Histiostoma* *feroniarum* | MF596167 | 13896 |
|  |  | Nothrina | Crotonioidea | Hermaniidae | *Hermannia* *gibba* | GEYB00000000.1 | 14255 |
|  |  |  |  | Crotoniidae | *Platynothrus* *peltifer* | GEYZ00000000.1 | 14915 |
|  |  |  |  | Nothridae | *Nothrus* *palustris* | GEYJ00000000.1 | 14551 |
|  |  | Brachypylina | Oripodoidea | Oribatulidae | *Oribatula* *sakamorii* | MT232643 | 14494 |
|  |  |  |  |  | *Oribatula* sp. | MH921998 | 15220 |
|  |  |  |  | Scheloribatidae | *Paraleius* *leontonychus* | LT984407 | 14186 |
|  | Mixonomatides |  | Phthiracaroidea | Phthiracaridae | *Steganacarus* *magnus* | EU935607 | 13818 |
| Trombidiformes | Eleutherengonides | Raphignathina | Tetranychoidea | Tetranychidae | *Tetranychus* *cinnabarinus* | NC_014399 | 13092 |
|  |  |  |  |  | *Tetranychus* *kanzawai* | KJ729017 | 13091 |
|  |  |  |  |  | *Tetranychus* *pueraricola* | KJ729021 | 13084 |
|  |  |  |  |  | *Tetranychus* *urticae* | NC_010526 | 13103 |
|  |  |  |  |  | *Tetranychus* *truncates* | KM111296 | 13089 |
|  |  |  |  |  | *Tetranychus* *ludeni* | KJ729018 | 13064 |
|  |  |  |  |  | *Tetranychus* *phaselus* | KJ729020 | 13084 |
|  |  |  |  |  | *Tetranychus* *malaysiensis* | KJ729019 | 13049 |
|  |  |  |  |  | *Amphitetranychus* *viennensis* | KX886344 | 13085 |
|  |  |  |  |  | *Panonychus* *citri* | HM189212 | 13077 |
|  |  |  |  |  | *Panonychus* *ulmi* | NC_012571 | 13115 |
|  |  |  | Cheyletoidea | Demodicidae | *Demodex* *brevis* | KM114225 | 14211 |
|  |  |  |  |  | *Demodex* *folliculorum* | KM114226 | 14150 |
|  | Parasitengonides | Hydracarina | Sperchontoidea | Sperchontidae | *Sperchon* *plumifer* | NC_039813 | 14646 |
|  | Eupodides |  | Eriophyoidea | Eriophyidae | *Phyllocoptes* *taishanensis* | KR604967 | 13475 |
|  |  |  |  |  | *Epitrimerus* *sabinae* | KR604966 | 13531 |
|  |  |  |  |  | *Leipothrix* sp. | KX027362 | 14216 |
|  |  |  |  | Diptilomiopidae | *Rhinotergum* *shaoguanense* | KX027361 | 13646 |
|  | Anystides | Parasitengonina | Trombiculoidea | Trombiculidae | *Leptotrombidium* *akamushi* | AB194045 | 13698 |
|  |  |  |  |  | *Leptotrombidium* *deliense* | AB194044 | 13731 |
|  |  |  |  |  | *Leptotrombidium* *pallidum* | AB180098 | 16779 |
|  |  |  |  |  | *Ascoschoengastia* sp. | NC_010596 | 16067 |
|  |  |  |  |  | *Walchia* *hayashii* | NC_010595 | 14857 |
|  |  |  | Hygrobatoidea | Unionicolidae | *Unionicola* *foili* | EU856396 | 14738 |
|  |  |  |  |  | *Unionicola* *parkeri* | HQ386015 | 14734 |
| Opiliones |  |  | Phalangioidea | Phalangiidae | *Phalangium* *opilio* | EU523757 | 14968 |
| Araneae |  |  | Araneoidea | Araneidae | *Neoscona* *theisi* | NC_026290 | 14156 |
| Ixodida |  |  | Ixodoidea | Argasidae | *Antricola* *mexicanus* | KC769591 | 14415 |
| Mesostigmata |  |  | Dermanyssoidea | Varroidea | *Varroa* *destructor* | AJ493124 | 16476 |
| Holothyrida |  |  | Holothyroidea | Allothyridae | *Allothyrus* sp. | KC769586 | 14578 |
| Pseudoscorpiones |  |  | Feaelloidea | Pseudogarypidae | *Pseudocellus* *pearsei* | NC_009985 | 15099 |
|  |  |  |  |  | *Pseudogarypus* *banksi* | JQ040544 | 16546 |
| Scorpiones |  |  | Buthoidea | Eremobatidae | *Eremobates* *c.f.palpisetulosus* | EU520642 | 15083 |
|  |  |  |  | Thelyphonidae | *Mastigoproctus* *giganteus* | EU520643 | 14416 |
|  |  |  |  | Buthidae | *Buthus* *occitanus* | EU523755 | 15060 |
| Xiphosura |  |  |  | Limulidae | *Carcinoscorpius* *rotundicauda* | JX437074 | 15037 |
|  |  |  |  |  | *Limulus* *polyphemus* | AF216203 | 14985 |
| Amblypygi |  |  |  | Phrynidae | *Phrynus* *sp.* | EU520641 | 14764 |
