# Supplementary material for: Mitochondrial analysis of oribatid mites provides insights into their atypical tRNA annotation, genome rearrangement and evolution
Source: Parasit Vectors. 2021 Apr 23;14:221. doi: 10.1186/s13071-021-04719-0 (PMC8063316; doi:10.1186/s13071-021-04719-0)
Supplement: Supplementary file 2 — Additional file 2: Table S2. Partition schemes used in the present study (DOCX 16 KB) [file 13071_2021_4719_MOESM2_ESM.docx]

**Table S2.** Partition schemes used in the present study

| **Dataset** | **number of**  **partitions** | **Analysis**  **method** | **Model and partition scheme** |
| --- | --- | --- | --- |
| Nucleotide  sequences | 20 | BI | **GTR+I+G** (ATP6_1^st^_2^nd^) (ATP8_1^st^_2^nd^, ND5_1^st^_2^nd^) (CYTB_1^st^_2^nd^) (COI_1^st^_2^nd^) (COII_1^st^_2^nd^) (COIII_1^st^_2^nd^) (ND1_1^st^_2^nd^) (ND2_1^st^_2^nd^) (ND3_1^st^_2^nd^) (ND4_1^st^_2^nd^, ND4L_1^st^_2^nd^) (ND6_1^st^_2^nd^ , ATP8_3^rd^) (COII_3^rd^) (ND1_3^rd^, ND4_3^rd^) (ND3_3^rd^, ND2_3^rd^)  **GTR+G** (ATP6_3^rd^, COIII_3^rd^) (CYTB_3^rd^) (COI_3^rd^) (ND4L_3^rd^) (ND5_3^rd^) (ND6_3^rd^) |
|  |  | ML | **GTRGAMMAI** (ATP6_1^st^_2^nd^) (ATP8_1^st^_2^nd^, ND5_1^st^_2^nd^) (CYTB_1^st^_2^nd^) (COI_1^st^_2^nd^) (COII_1^st^_2^nd^) (COIII_1^st^_2^nd^) (ND1_1^st^_2^nd^) (ND2_1^st^_2^nd^) (ND3_1^st^_2^nd^) (ND4_1^st^_2^nd^, ND4L_1^st^_2^nd^) (ND6_1^st^_2^nd^ , ATP8_3^rd^) (COII_3^rd^) (ND1_3^rd^, ND4_3^rd^) (ND3_3^rd^, ND2_3^rd^) (ATP6_3^rd^, COIII_3^rd^) (CYTB_3^rd^) (COI_3^rd^) (ND4l_3^rd^) (ND5_3^rd^) (ND6_3^rd^) |
|  | 8 | BI | **GTR+I+G** (ATP6_1^st^_2^nd^) (ND6__1^st^_2^nd^, ATP8_1^st^_2^nd^) (COII_1^st^_2^nd^, CYTB_1^st^_2^nd^) (COII_1^st^_2^nd^) (COIII_1^st^_2^nd^) (ND5_1^st^_2^nd^, ND1_1^st^_2^nd^) (ND1_1^st^_2^nd^, ND3_1^st^_2^nd^) (ND4_1^st^_2^nd^, ND4l_1^st^_2^nd^) |
|  |  | ML | **GTRGAMMAI**(ATP6_1^st^_2^nd^) (ND6__1^st^_2^nd^, ATP8_1^st^_2^nd^) (COII_1^st^_2^nd^, CYTB_1^st^_2^nd^) (COII_1^st^_2^nd^) (COIII_1^st^_2^nd^) (ND5_1^st^_2^nd^, ND1_1^st^_2^nd^) (ND1_1^st^_2^nd^, ND3_1^st^_2^nd^) (ND4_1^st^_2^nd^, ND4l_1^st^_2^nd^) |
| Amino acid | 1 | BI | **LG+G**(ATP6_1^st^_2^nd^_3^rd^) (ND3_1^st^_2^nd^_3^rd^, ND4_1^st^_2^nd^_3^rd^)  **LG+I+G** (CYTB_1^st^_2^nd^_3^rd^) (COI_1^st^_2^nd^_3^rd^) (COII_1^st^_2^nd^_3^rd^, ND1_1^st^_2^nd^_3^rd^) (COIII_1^st^_2^nd^_3^rd^, ND5_1^st^_2^nd^_3^rd^) (ND2_1^st^_2^nd^_3^rd^) |
|  |  | ML | **GTRGAMWAG** (ATP6_1^st^_2^nd^_3^rd^) (ND3_1^st^_2^nd^_3^rd^, ND4_1^st^_2^nd^_3^rd^) (CYTB_1^st^_2^nd^_3^rd^) (COI_1^st^_2^nd^_3^rd^) (COII_1^st^_2^nd^_3^rd^, ND1_1^st^_2^nd^_3^rd^) (COIII_1^st^_2^nd^_3^rd^, ND5_1^st^_2^nd^_3^rd^)( ND2_1^st^_2^nd^_3^rd^) |
