# Supplementary material for: Mitochondrial analysis of oribatid mites provides insights into their atypical tRNA annotation, genome rearrangement and evolution
Source: Parasit Vectors. 2021 Apr 23;14:221. doi: 10.1186/s13071-021-04719-0 (PMC8063316; doi:10.1186/s13071-021-04719-0)
Supplement: Supplementary file 3 — Additional file 3: Table S3. Mitochondrial genome organization of Oribatula sakamorii (DOCX 20 KB) [file 13071_2021_4719_MOESM3_ESM.docx]

| Gene | Start | End | Strand | Length | Gap | Start codon | Stop codon |
| --- | --- | --- | --- | --- | --- | --- | --- |
| *cox1* | 1 | 1581 | + | 1581 | 2 | ATT | TAG |
| *cox2* | 1584 | 2249 | + | 666 | -2 | ATG | TAA |
| *trnD* | 2248 | 2303 | + | 56 | -1 |  |  |
| *atp8* | 2303 | 2452 | + | 150 | -4 | ATA | TAA |
| *atp6* | 2449 | 3114 | + | 666 | 0 | ATA | TAA |
| *cox3* | 3115 | 3900 | + | 786 | -1 | ATC | TAA |
| *trnG* | 3900 | 3953 | + | 54 | 0 |  |  |
| *nad3* | 3954 | 4295 | + | 342 | 12 | ATA | TAA |
| *trnA* | 4308 | 4357 | + | 50 | 2 |  |  |
| *trnL2* | 4360 | 4418 | + | 59 | -4 |  |  |
| *trnL1* | 4415 | 4475 | + | 61 | -2 |  |  |
| *trnS1* | 4474 | 4526 | + | 53 | 24 |  |  |
| *trnF* | 4551 | 4610 | - | 60 | 10 |  |  |
| *nad5* | 4621 | 6240 | - | 1620 | 0 | ATG | TAA |
| *trnH* | 6241 | 6299 | - | 59 | -2 |  |  |
| *nad4* | 6298 | 7593 | - | 1296 | 1 | ATG | TAA |
| *nad4L* | 7595 | 7864 | - | 270 | -1 | ATT | TAA |
| *trnT* | 7864 | 7919 | + | 56 | 0 |  |  |
| *nad6* | 7920 | 8345 | + | 426 | -1 | ATA | TAA |
| *cob* | 8345 | 9439 | + | 1095 | -1 | ATA | TAA |
| *trnS2* | 9439 | 9493 | + | 55 | -3 |  |  |
| *nad1* | 9491 | 10387 | - | 897 | 52 | ATG | TAG |
| *trnM* | 10440 | 10495 | + | 56 | -1 |  |  |
| *trnW* | 10495 | 10554 | + | 60 | -4 |  |  |
| *trnC* | 10551 | 10602 | - | 52 | 4 |  |  |
| *nad2* | 10607 | 11560 | + | 954 | -2 | ATT | TAA |
| *trnI* | 11559 | 11615 | + | 57 | 2 |  |  |
| *trnQ* | 11618 | 11677 | - | 60 | -4 |  |  |
| *trnY* | 11674 | 11724 | - | 51 | -5 |  |  |
| *trnP* | 11720 | 11772 | - | 53 | 0 |  |  |
| *rrnL* | 11773 | 12842 | - | 1070 | 0 |  |  |
| *trnV* | 12843 | 12890 | - | 48 | 0 |  |  |
| *rrnS* | 12891 | 13572 | - | 682 | 0 |  |  |
| *trnN* | 13573 | 13628 | - | 56 | -3 |  |  |
| *trnR* | 13626 | 13669 | + | 44 | 3 |  |  |
| *trnE* | 13673 | 13733 | + | 61 | 11 |  |  |
| *trnK* | 13745 | 13809 | + | 65 | 0 |  |  |
| CR | 13810 | 14494 | + | 685 | 0 |  |  |

**Table S3**. Mitochondrial genome organization of *Oribatula* *sakamori*
