# Supplementary figures and images for: Mitochondrial analysis of oribatid mites provides insights into their atypical tRNA annotation, genome rearrangement and evolution
Source: Parasit Vectors. 2021 Apr 23;14:221. doi: 10.1186/s13071-021-04719-0 (PMC8063316; doi:10.1186/s13071-021-04719-0)

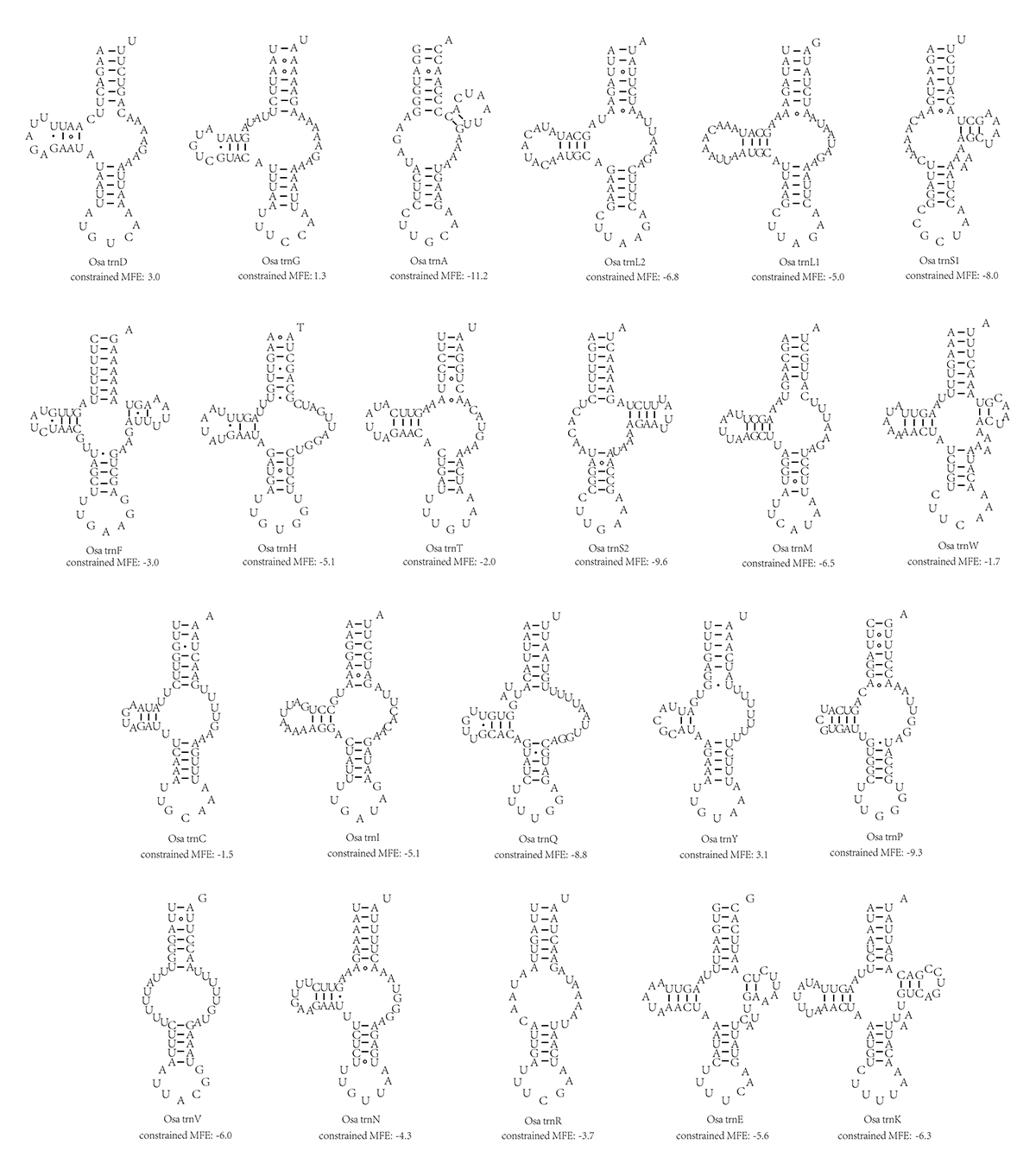

Supplement: Supplementary file 4 — Additional file 4: Figure S1. Secondary structures of tRNAs of Oribatula sakamorii (Osa). The tRNA short name and the calculated constrained MFE is indicated (TIF 274 KB) [file 13071_2021_4719_MOESM4_ESM.tif]
